# Assessing Coverage, Equity and Quality Gaps in Maternal and Neonatal Care in Sub-Saharan Africa: An Integrated Approach

**DOI:** 10.1371/journal.pone.0127827

**Published:** 2015-05-22

**Authors:** Calistus Wilunda, Giovanni Putoto, Donata Dalla Riva, Fabio Manenti, Andrea Atzori, Federico Calia, Tigist Assefa, Bruno Turri, Onapa Emmanuel, Manuela Straneo, Firma Kisika, Giorgio Tarmbulini

**Affiliations:** 1 Projects Department, Doctors with Africa CUAMM, Padua, Italy; 2 Department of Pharmacoepidemiology, Graduate School of Medicine and Public Health, Kyoto University, Kyoto, Japan; 3 Doctors with Africa CUAMM, Wolisso, Ethiopia; 4 WolissoHospital, Wolisso, Ethiopia; 5 Doctors with Africa CUAMM, Aber, Uganda; 6 Aber Hospital, Aber, Uganda; 7 Doctors with Africa CUAMM, Iringa, Tanzania; 8 Ministry of Health and Social Welfare, Iringa, Tanzania; 9 European School for Maternal, Newborn, Child and Adolescent Health and Centro per la Salute del Bambino, Trieste, Italy; The Foundation for Medical Research, INDIA

## Abstract

**Background:**

Gaps in coverage, equity and quality of health services hinder the achievement of the Millennium Development Goals 4 and 5 in most countries of sub-Saharan Africa as well as in other high-burden countries, yet few studies attempt to assess all these dimensions as part of the situation analysis. We present the base-line data of a project aimed at simultaneously addressing coverage, equity and quality issues in maternal and neonatal health care in five districts belonging to three African countries.

**Methods:**

Data were collected in cross-sectional studies with three types of tools. Coverage was assessed in three hospitals and 19 health centres (HCs) utilising emergency obstetric and newborn care needs assessment tools developed by the Averting Maternal Death and Disability program. Emergency obstetrics care (EmOC) indicators were calculated. Equity was assessed in three hospitals and 13 HCs by means of proxy wealth indices and women delivering in health facilities were compared with those in the general population to identify inequities. Quality was assessed in three hospitals using the World Health Organization’s maternal and neonatal quality of hospital care assessment tool which evaluates the whole range of aspects of obstetric and neonatal care and produces an average score for each main area of care.

**Results:**

All the three hospitals qualified as comprehensive EmOC facilities but none of the HCs qualified for basic EmOC. None of the districts met the minimum requisites for EmOC indicators. In two out of three hospitals, there were major quality gaps which were generally greater in neonatal care, management of emergency and complicated cases and monitoring. Higher access to care was coupled by low quality and good quality by very low access. Stark inequities in utilisation of institutional delivery care were present in all districts and across all health facilities, especially at hospital level.

**Conclusion:**

Our findings confirm the existence of serious issues regarding coverage, equity and quality of health care for mothers and newborns in all study districts. Gaps in one dimension hinder the potential gains in health outcomes deriving from good performances in other dimensions, thus confirm the need for a three-dimensional profiling of health care provision as a basis for data-driven planning.

## Introduction

Most countries in sub-Saharan Africa (SSA) lag behind in achievement of the Millennium Development Goals (MDGs) 4 and 5 [1, 2] due to complex, and variable across countries, combination of social and health system factors [3, 4]. Among the latter, gaps in coverage, equity and quality of maternal, neonatal and child health services are of paramount importance. Coverage gaps along the continuum of care have long been identified [5] and addressed, with variable results, by nation-wide and internationally driven plans and programs [6]. Dramatic inequities have been acknowledged [7] and are now being increasingly addressed in strategic documents, action plans and related indicators, although so far seldom translated into concrete actions. The importance of the quality gap has been recognized only recently by international agencies and it is now gradually being given greater importance [8, 9].

Indeed, increased coverage of maternal and neonatal health interventions usually results into reduced inequity [10], but it is unlikely to reduce maternal and child deaths if the quality of care rendered is poor [11]. On the other hand, the impact of quality improvement on maternal and newborn health outcomes may be low if access to the service is poor and those at higher risk are excluded [12]. International and national plans and programs, while acknowledging the existence of gaps in all three dimensions of health care provision, tend to focus on each of them in isolation. A health system framework has been proposed to identify bottlenecks for neonatal care [13], yet an integrated approach to the assessment of coverage, equity and quality gaps as a basis for a data-driven planning process is still lacking.

Within a non-governmental organisation (NGO)-run large project aimed at improving maternal and neonatal health in five districts in three SSA countries, a simultaneous assessment of coverage, equity and quality was carried out to inform priority setting and planning. We report on the methods and results of such an approach and discuss its potential use in other high-burden countries.

## Materials and Methods

### Institutional background

Doctors with Africa CUAMM (hereafter referred to as CUAMM) is an Italian NGO which has been supporting health service delivery in Africa for over 60 years. The organisation has adopted the continuum of care approach as the main health service delivery strategy in its interventions [14]. In 2012, CUAMM started to implement a five-year project focusing on equitable and effective access to safe childbirth and neonatal health services (dubbed “mothers and children first”) in three SSA countries: Ethiopia, Tanzania and Uganda. The project aims at achieving improved coverage and quality of essential maternal and neonatal health services and reduced inequity in access to the services.

### Settings

Three countries are involved in the project: Ethiopia, Tanzania and Uganda. All three countries belong to the high mortality (both maternal and under 5), high priority group of countries and are included in internationally driven strategies and initiatives such as the United Nations Secretary General’s Strategy [15] and the countdown to 2015 initiative [16], being among the five countries that account for half of Africa’s newborn deaths [17]. The project is being conducted in Wolisso, Goro and Wonchi (WGW) districts in Ethiopia, Oyam district in Uganda and Iringa district in Tanzania. All the districts and related health facilities covered by the project in the countries were included in the study.

In order to accelerate reduction in maternal and neonatal mortality, government guidelines in the study countries require all health centres (HCs) and hospitals to provide basic emergency obstetric care (BEmOC) and comprehensive emergency obstetric care (CEmOC), respectively [18–20].

Table 1 presents the main demographic and health system indicators of the three countries and five districts included in the study. All three district hospitals are private-not-for-profit, belong to national health systems, and are supported by CUAMM through provision of expatriate health professionals and variable amounts of financial support.

**Table 1:** Main demographic and health system features of the three countries and five districts included in the study.

Study countries			
Feature	Ethiopia	Tanzania	Uganda
Maternal mortality ratio per 100,000 live births^a^	676	454	438
Neonatal mortality rate per 1,000 live births^a^	37	26	27
Stillbirth rate per 1,000 births^b^	25.6	25.6	24.8
Health expenditure per capita (US$-in purchasing power parity)^c^	52	107	128
National health worker ratio per 10,000 population^d^	3	3	14
**Study districts**			
**Feature**	**Wolisso, Goro & Wonchi districts**	**Iringa district**	**Oyam district**
Population (2011)	372,533	276,809	378,900
Expected number of births	13,895	10,796	18,377
Institutional delivery coverage	20% ^e^	90%^f^	42% ^e^
Number of hospitals	1	1	1
Number of health centres (HC III & IV in Uganda)	16	6	6
Number of dispensaries/health posts (HC II in Uganda)	89	59	17

^a^ Source: latest demographic and health survey

^b^ Source: WHO data on country stillbirth rates per 1000 total births for 2009.

^d^ Doctors, nurses and midwives; the recommended number is 23.

^C^ Source: 2014 WHO African Region country statistics summary (2002—present).

^e^ Based on data from the health management information system

^f^ District household survey

### Data collection tools and methods

A summary of tools and methods of data collection is presented in Table 2. Three types of tools were used to collect cross-sectional baseline data on coverage, equity and quality. With respect to coverage, both availability and actual use of services were assessed using the “needs assessment of emergency obstetric and newborn care” (EmONC) tools developed by the Averting Maternal Death and Disability (AMDD) program of Columbia University [21]. The AMDD’s EmONC needs assessment toolkit consists of 10 modules that cover various aspects of the health system including health facility infrastructure, human resources, drugs; equipment and supplies, facility statistics, availability of emergency obstetrics care (EmOC) signal functions, provider knowledge and competency in maternal and newborn care and the referral system. The findings in this paper are based on modules 4 (facility case summary) and 5 (EmOC signal functions). The facility case summary module was completed by reviewing hospital registers and monthly summary sheets. Data on EmOC signal functions were collected at each health facility by interviewing the maternity ward in-charge using the EmOC signal functions module. To improve the accuracy of the data, data collectors, after specific ad hoc training, reviewed several records to double-check the data.

**Table 2:** A summary of the tools and methodology of data collection.

Assessment	Tools	Data collection methods	Data collectors	Facilities involved
Coverage	Modules 4 and 5 of emergency obstetric and newborn care needs assessment tools by Averting Maternal Death and Disability program	Review of health facility statistics and medical records; interviews with the maternity ward in-charge staff.	Nurses and midwives working in the district and specifically trained.	3 hospitals and 19 health centres
Equity	Proxy wealth indices developed and validated using Demographic and Health Survey data	Interviews with mothers after delivery	Nurses and midwives working in the maternity ward of study facilities	3 hospitals and 13 health centres
Quality	World Health Organization’s maternal and neonatal quality of hospital care assessment tool	Review of hospital statistics, medical records, direct observation of cases, and semi-structured interviews with staff and mothers.	A team consisting of an obstetrician, a midwife and a paediatrician/neonatologist	3 hospitals

Data from these two modules were used to calculate EmOC indicators according to the standard United Nations guidelines [22] as summarised in S1 Table. EmOC indicators were used to measure the availability, use and, to a limited extent, the quality of maternal and neonatal health services. The EmOC status of a health facility is defined based on whether the facility performed certain signal functions in a three-month period prior to the survey. A BEmOC facility is one that performed all of the following 7 signal functions: i) administration of parenteral antibiotics; ii) administration of oxytocic drugs; iii) administration of anticonvulsants; iv) manual removal of placenta; v) removal of retained products; vi) assisted vaginal delivery; and vii) neonatal resuscitation with bag and mask. A CEmOC facility is one which performed all BEmOC signal functions as well as caesarean sections and blood transfusions [22]. In calculating the proportion of births in EmOC facilities, the met need for EmOC and the caesarean section coverage, we excluded 35.6% and 11% women from the neighbouring districts who sought treatment in the study hospital in Ethiopia and Uganda, respectively. However, we did not exclude women resident in the study districts who might have been treated in neighbouring districts because their number, due to long distances, was negligible. Data from Tanzania were not detailed enough to allow this kind of adjustment. We collected data on coverage between August and November 2012 from one hospital and seven HCs in Ethiopia, and from one hospital and six HCs in each of Uganda and Tanzania.

We assessed equity using proxy wealth indices developed according to the methodology proposed by Pitchforth *et al*. [23] and previously applied to one of the participating hospitals [24]. In brief, the methodology consists of three steps: 1) using household survey data to select a small set of proxy wealth variables; 2) developing a questionnaire using the selected wealth variables; and 3) using the questionnaire to compare the wealth status of women utilising delivery services with that of women in the general population and thereby identifying inequity in the former group. A detailed description can be found in the original papers [23, 24]. The main features of the equity surveys are as summarised in S2 Table. We developed the equity assessment questionnaires based on Demographic and Health Survey (DHS) data of the respective countries. For each country, we selected 6 out of about 40 variables from the DHS; we attributed scores to these variables and assessed the validity and reliability of the scores using Pearson’s correlation coefficient (rho) and a measure of agreement (kappa), respectively, with reference to the DHS wealth index. Details regarding the scoring system are available in S3 Table. The selection of only 6 variables was driven by the need to have a simple and easy to administer tool that will cause minimal inconvenience to respondents, yet valid and reliable enough to measure the socio-economic status of the service users. The selected variables were then included in a one-page questionnaire and pretested. Two midwives/nurses were recruited at each health facility and trained on data collection. The data collectors invited all women who had delivered at the health facility to participate in the interview. Equity assessment was conducted between December 2011 and February 2013.

In Uganda and Ethiopia, we collected data at both the hospital and HCs whilst in Tanzania we collected data only at the hospital. This was because a previous household survey in the Iringa District in Tanzania had shown that coverage for institutional delivery was very high (90%) and equitable, but there was some concern that utilisation of the highest level of care, i.e. at hospital, was inequitable.

Data were analysed using Stata version 11. Scores from proxy wealth variables for each woman were summed up and were used to categorize women into wealth groups using the cut-off points for wealth quintiles of women in the household survey data. In the household survey data women are equally distributed in the wealth quintiles (about 20% in each quintile) and any significant shift from this distribution among users of service implies inequity. We compared the household survey data with our collected data using F tests (chi squared tests adjusted for survey design) [25]. In doing so, we used Stata commands that account for the complex sampling design and weighting used in DHS.

Quality was assessed using the World Health Organization’s maternal and neonatal quality of hospital care assessment tool [26]. The tool is standard-based and action-oriented and assesses the whole range of obstetric and neonatal care across 17 areas from support services to case management, focusing primarily on safety and effectiveness but also on women’s rights to respect, confidentiality and information. More than 400 items were assessed. Four sources of information were utilised: hospital statistics, medical records, direct observation of cases, and semi-structured interviews with staff and mothers. Interviews with staff were mainly aimed at exploring knowledge and use of guidelines, organizational procedures and team work. Interviews with mothers explored obstacles to access and patients’ satisfaction with the care and information received. A minimum of ten staff members and ten mothers was interviewed in each hospital. The sample included a variety of women who had experienced either vaginal deliveries or caesarean sections. Mothers with premature babies admitted to the neonatal ward and mothers with babies readmitted to hospital were also represented in the sample.

By combining the information from the various sources, scores ranging from 3 to 0 were attributed to each item based on the following criteria: 3 = care corresponding to international standards (no need for improvement or need for marginal improvement); 2 = substandard care but no serious hazard to health or violation of human rights; 1 = inadequate care with consequent serious health hazards or violation of women’s rights to information, privacy or confidentiality and/or to children’s rights; 0 = very poor care with consequent systematic and severe hazards to the health of mothers and/or newborns, e.g. systematic omission of potentially life-saving interventions or lack of essential safety requisites for key procedures such as caesarean section, blood transfusion, neonatal resuscitation, etc. By summing up all scores, an overall average score for each main area of care was obtained. The assessments were conducted by an external multidisciplinary team (an obstetrician, a midwife, and a paediatrician/neonatologist) and involved hospital managers and health professionals. The assessments led to identification of main gaps in quality of care and to a draft plan of actions which included all issues amenable to change based on hospital resources. To ensure consistency of the assessment process, the assessment teams followed the standardized methods described for the tool [26], and one team member participated in all three assessments. Moreover, most team members had previously conducted such assessments jointly in other countries [27]. Quality assessments were conducted between August and October 2012 in all three participating hospitals.

### Ethics statement

Ethical approvals to conduct the studies were obtained from the Oromiya Regional Institutional Review Committee in Ethiopia, the National Council for Science and Technology in Uganda and the National Institute of Medicine in Tanzania. The studies were also approved by the respective district health management teams in each participating district. Participants in the equity and quality studies provided signed informed consent after the objectives and methods of the study had been explained to them. Those who could not write provided verbal consent in the presence of a witness. Coverage and quality assessments relied mainly on observation and review of routine health data and medical records hence did not require informed consent. Verbal consent in the presence of a witness was obtained from interviewed mothers. All collected data were anonymous and did not contain any information that might be used to identify individual patients.

## Results

### Coverage

**Fig 1:**
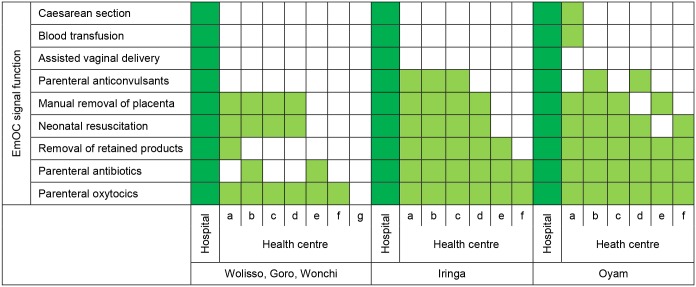
Performance of EmOC signal functions in a three-month period prior to the survey.

Table 3 presents a summary of EmOC indicators in the five districts. Regarding the availability of EmOC, all three hospitals performed the nine EmOC signal functions in a three-month period prior to the survey and hence qualified as CEmOC centres. In contrast, none of the 19 HCs performed all the required seven EmOC signal functions to qualify as a BEmOC centre (Fig 1). In Uganda, one HC (a HC level IV) was performing caesarean sections and transfusing blood, but was not able to perform two BEmOC signal functions.

**Table 3:** Emergency obstetric care (EmOC) indicators in the study districts.

EmOC indicator	Acceptable standard	Wolisso, Goro & Wonchi (Ethiopia)	Oyam (Uganda)	Iringa (Tanzania)
1a. Number of comprehensive EmOC facilities per 500,000 population	1	1	1	1.8
1b. Number of basic EmOC facilities per 500,000 population	4	0	0	0
2. Proportion of all births in EmOC facility	To be set locally	13.4% (1868/13895)	8.6% (1584/18377)	18.0% (1942/10796)
3. Met need for EmOC services	100%	17.1% (356/2084)	13.3% (366/2757)	19.5% (316/1619)
4. Caesarean sections as a proportion of all births	5–15%	2.3% (315/13895)	2.1% (390/18377)	5.5% (594/10796)
5. Direct obstetric case fatality rate in EmOC facility	<1	0.9% (5/553)	1.5% (6/411)	1.6% (5/316)
6. Intrapartum and very early neonatal death rate in EmOC facility	None set	1.0% (30/2900)	2.3% (41/1780)	1.5% (29/1942)
7. Proportion of maternal deaths due to indirect causes in EmOC facility	None set	16.7% (1/6)	33.3% (3/9)	37.5% (3/8)

The three districts in Ethiopia showed the largest gap in the provision of EmOC signal functions in HCs. Overall, the biggest gaps were found in the provision of assisted vaginal delivery (AVD) using vacuum or forceps and administration of anticonvulsants for preeclampsia/eclampsia management. No HC was able to perform AVD and only 5 out of 19 HCs had administered anticonvulsants three months prior to the survey. The main reasons why HCs were not able to provide the EmOC signal functions were lack of equipment and supplies, inadequate training and lack of indication as summarised in S4 Table. None of the five districts met the United Nations’ (UN) standard of 1 CEmOC and 4 BEmOC facilities per 500,000 inhabitants.

With respect to the actual use of services, the proportion of births occurring in a health facility was 20% in WGW districts in Ethiopia, 42%in Oyam district in Uganda and 90% in Iringa rural district in Tanzania.

The proportion of births occurring in EmOC facilities, was lowest in Oyam district (8.6%) and highest (18%) in Iringa rural district. The met need for EmOC was less than 20% across all districts. Caesarean section rate in Iringa district (5.5%) was within the recommended range, while in other districts, it was below the minimum recommended rate.

Regarding maternal and perinatal outcomes, the lowest direct obstetric case-fatality rate in EmOC facilities was reported in WGW (0.9% each) and the highest was reported in Iringa (1.6%). Combined intrapartum and very early neonatal death rate in EmOC facilities ranged between 1% in WGW and 2.3 in Oyam. In non-EmOC facilities, this indicator was 0.5% in both WGW and Oyam and 0.2% in Iringa. The number of deaths at HCs was small since most complicated cases are referred to hospital before they die. The highest proportion of maternal deaths due to indirect causes in EmOC facilities was found in Iringa (37.5%) and the lowest in WGW (16.7%); reflecting the prevalence of HIV/AIDS across the districts: 15% in Iringa, 11% in Oyam and 2% in WGW.

### Equity

**Fig 2:**
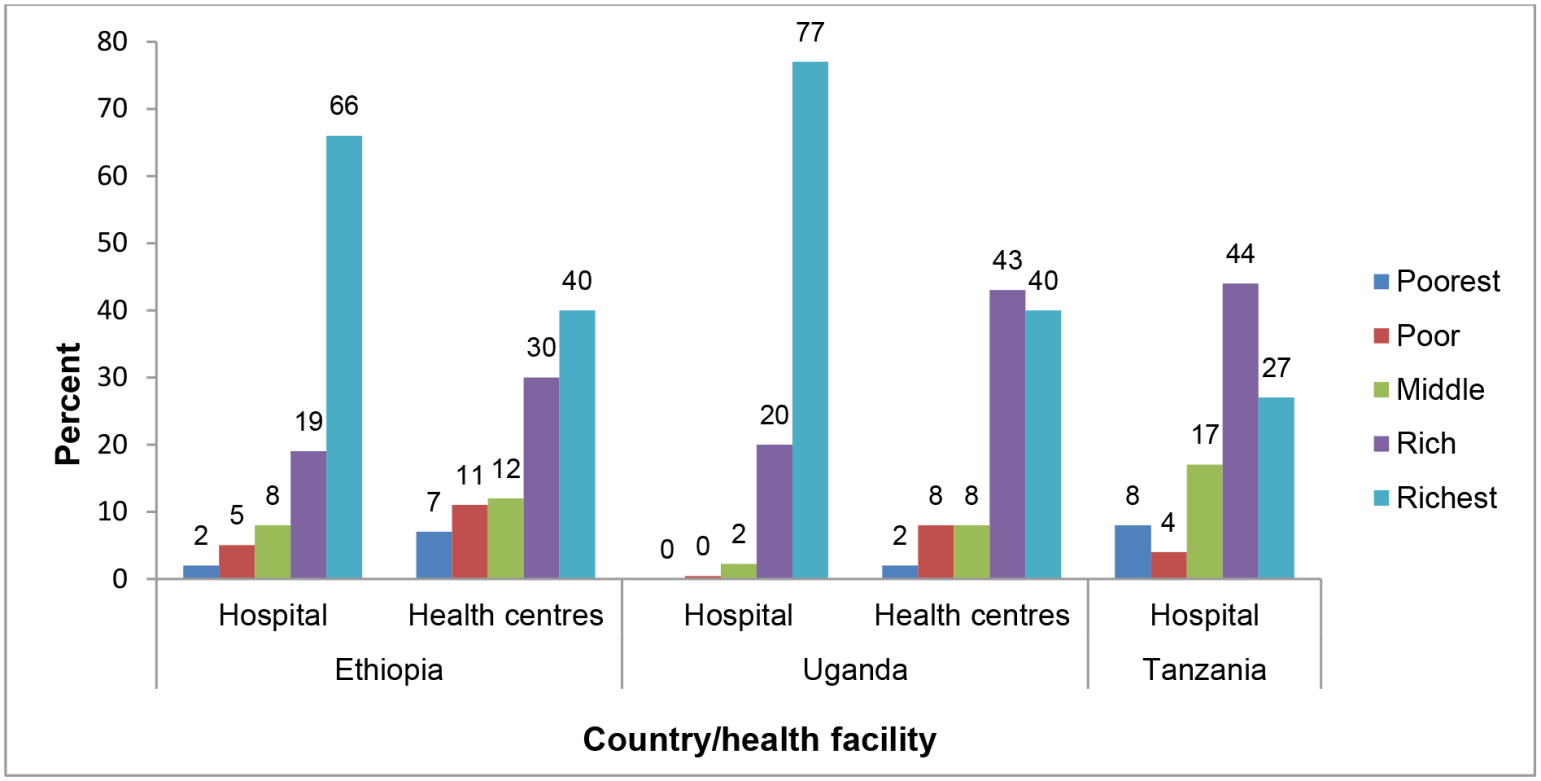
Distribution of women delivering at health facilities in the study districts into wealth groups.

Data on equity was collected from a total of 3,643 women: 1,285 in WGW (response rate 94%), 1,354 in Oyam (response rate 81%) and 986 in Iringa (response rate 99%). Fig 2 shows the proportion of women having access to institutional delivery in study districts by wealth quintiles and health facility level (for WGW and Oyam). Data from our study show that in WGW districts, 66% of women who delivered at hospital belonged to the richest wealth group while only 2% belonged to the poorest wealth group. In the same districts, 40% of those who delivered at HCs belonged to the richest group while 7% belonged to the poorest group. Among users of delivery services at the hospital in Oyam, 77% belonged to the richest wealth group whilst less than 1% belonged to the poorest two wealth groups. At HCs, the situation was slightly better as 40% of users belonged to the richest group and 2% belonged to the poorest group. Iringa District was the least inequitable. Overall, the poorest population group was strikingly underrepresented across all the districts (Table 4).

**Table 4:** Percent distribution of women utilising delivery services and those in the general population in the study districts, by wealth group.

Wealth group	Wolisso, Goro and Wonchi			Oyam			Iringa		
	DHS^a^ (n = 1447)	Delivery service users (n = 1305)	P value^d^	DHS^b^(n = 1051)	Delivery service users (n = 1352)	P value^d^	DHS^c^ (n = 341)	Delivery service users (n = 986)	P Value^d^
Poorest	19	3	<0.001	20	1	<0.001	19	8	<0.001
Poor	21	6		20	5		24	4	
Middle	21	8		20	6		17	17	
Rich	20	20		19	35		17	44	
Richest	19	63		21	52		23	27	

^a^ the 2011 Ethiopia Demographic and Health Survey data of Oromiya Region

^b^ the 2006 Uganda Demographic and Health Survey data of Northern Uganda

^c^ the 2010Tanzania Demographic and Health Survey data of Iringa Region

^d^ P value from an F test which is a chi squared test adjusted for the complex sampling design used in demographic and health surveys

### Quality

**Fig 3:**
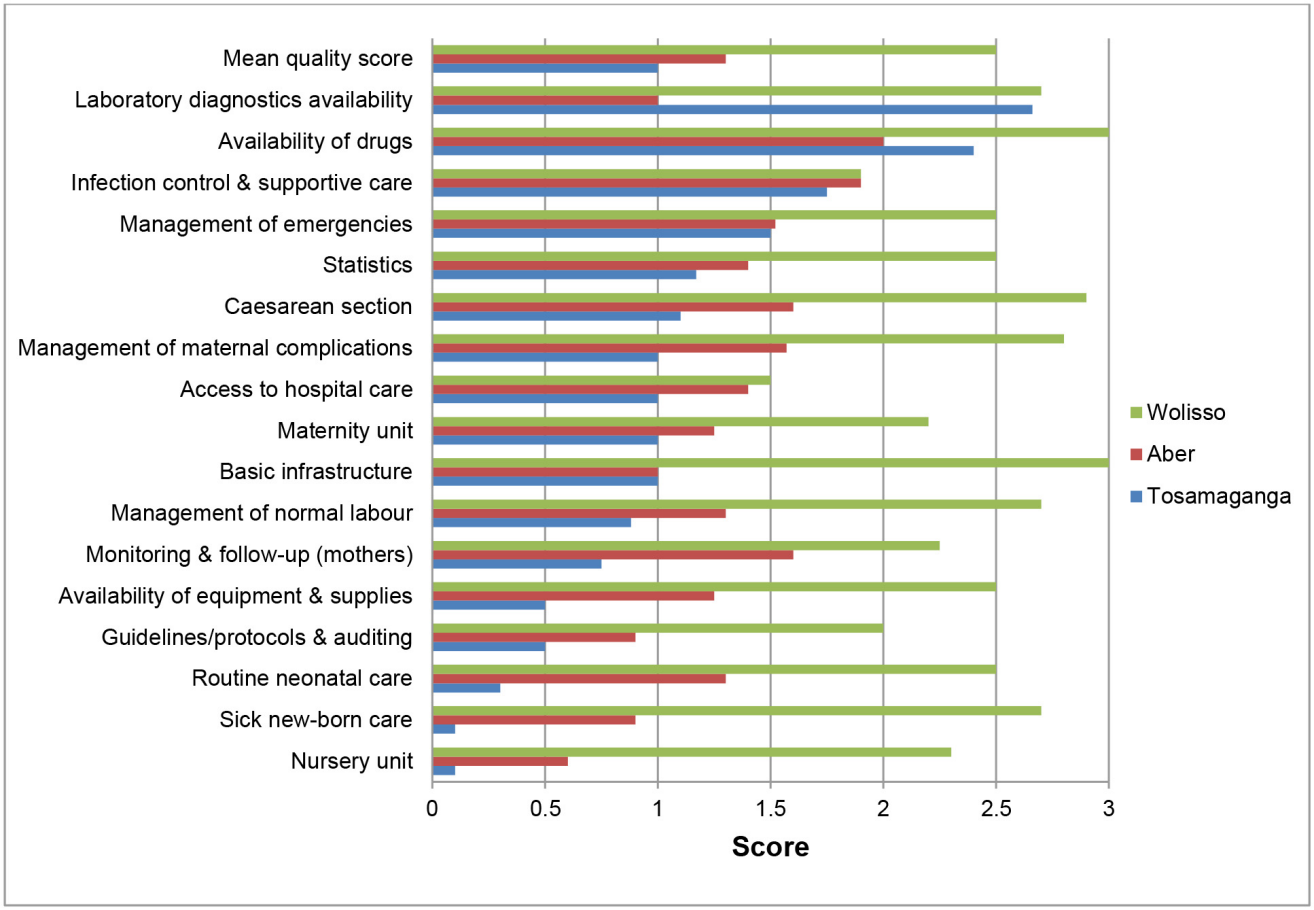
Quality of maternal and new-born care services at Wolisso (Ethiopia), Aber (Uganda) and Tosamaganga (Tanzania) hospitals.

The distribution of scores in the main areas included in the assessment of quality of care conducted in the three hospitals belonging to the study districts is shown in Fig 3. Overall, Wolisso hospital had the highest average quality score (2.5/3) while Tosamaganga hospital had the lowest (1.0/3). In two out of three hospitals, important and widespread quality gaps were shown in all 17 areas, with partial exceptions for laboratory equipment and availability of drugs. According to the definitions used, gaps were severe enough to imply serious (score below 2) or systematic and severe (score below 1) hazards to the health of mothers and/or newborns. Overall, gaps were generally greater in neonatal care-which was virtually absent in one hospital, and in other key aspects such as, management of normal labour and obstetric complications. Protocols and monitoring procedures were also lacking in two out of three hospitals. The situation was significantly better in the hospital in Ethiopia where a similar assessment, mainly focused on neonatal care, was carried out about 18 months earlier, leading to substantial improvements in the care of the normal newborn and in the case management of low birth weight babies. Quality of care at hospital level was reflected in case fatality rates. Besides and beyond safety and effectiveness issues, complaints by mothers included mainly the following: inadequate bath or shower facilities; restricted entry of family members and relatives; staff sometimes not providing enough information or not responding to specific requests (e.g. for pain relief); and lack of respect by staff, including cleaners.

### Summary of three-dimension assessment

As shown in Table 5, in none of the districts, both sufficient access and satisfactory quality were ensured. Where good quality of care was provided at hospital level, as in Wolisso Hospital, access to BEmOC facilities was very low at district level. Where access to institutional delivery was high, as in Oyam district in Tanzania, quality of care provided at hospital level was low. The overall score of quality of care was inversely related to case fatality rate at hospital level.

**Table 5:** Overview of main coverage, equity and quality indicators.

Indicator		District		
		Wolisso, Goro & Wonchi	Oyam	Iringa
Coverage				
	Met need for EmOC	17.1%	13.3%	19.5%
	Caesarean section coverage	2.3	2.1%	5.5%
Equity				
	Percent of women in the lowest two wealth groups	9%	6%	12%
	Percent of women in the highest two wealth groups	83%	87%	71%
Quality				
	Mean quality score (out of 3)	2.5	1.3	1.0
	Direct obstetric case fatality rate	0.9%	1.5%	1.6%

### Action planning

To address the problem of poor availability of EmOC, CUAMM in consultation with district health authorities started a process of supporting the upgrading of HCs to attain BEmOC status in all districts. This included: infrastructural improvements; procurement and supply of missing equipment, supplies and drugs; training of staff; strengthening the referral system including provision of free ambulance services and regular supervision of HCs. Improvement of health information systems to collect high quality data to monitor EmOC indicators was also planned. To improve coverage and address inequity, user fees for maternal complications, including caesarean sections, were removed in all hospitals. Because the EmOC status of a health facility depends on its utilisation, a community mobilisation strategy to increase service use was developed. Additionally, a strategy of geographical targeting was designed whereby poorer and underserved areas of the districts were targeted with demand-side incentives to improve access and use. The incentive package consisted of transport vouchers, supply of baby kits and free motor cycle ambulance for women who choose to deliver in health facilities. Action plans to improve the quality of care by addressing the main gaps identified by the assessment were developed in all three hospitals, starting with a list of priority actions recommended by the assessment team and discussed at the debriefing session after the assessment, and later on finalized and completed. A follow-up assessment was planned within 24 months in both Aber and Tosamaganga hospitals, while in Wolisso, where results of the assessment were satisfactory, only an internal follow-up assessment was planned.

## Discussion

This is to our knowledge the first action-oriented attempt to collect baseline data on coverage, equity and quality of maternal and newborn care in a multi-country sample of health facilities. The approaches that were used to assess these three dimensions of health care proved to be feasible within a relatively limited period of time and were sufficiently informative to provide a detailed snapshot of the existing challenges in all three dimensions, thus providing a model that could be replicated in other high-burden countries.

Our findings confirm the existence of important gaps in all three dimensions of health care, and the need to address them simultaneously since gaps in one dimension hinder the potential gains in health outcomes deriving from advances and satisfactory performances in other dimensions. The situations we have described are illuminating in this respect. In WGW districts in Ethiopia, where only 20% of women have access to institutional delivery, HCs are not yet able to provide EmOC and hospital care is strikingly restricted to the better-off, and so are the benefits of a generally good quality of care at hospital level. Here the priority is clearly to improve both demand for and provision of antenatal and delivery care, by increasing the number of BEmOC centres and by addressing accessibility issues such as distance. In Uganda, where 42% of women have access to institutional delivery care, further efforts are needed to increase access across all population groups and to improve the quality of care provided at facility level. In Tanzania, where access is high but HCs do not qualify as BEmOC and the quality is very low even at the district hospital, investing in quality is the outstanding priority. The availability of enough CEmOC facilities but a shortage of BEmOC facilities reported in this study appear to be a common finding in many EmOC surveys [28, 29].

Our estimates of met need for EmOC across the three countries are in line with the results of a recent review which has found that the met need for EmOC in low income countries is 21% (interquartile range of 12–31%) [30]. Although the low met need for EmOC is a reflection of poor access to maternal health care, it is also partly due to exclusion from the calculation of some women with obstetric complications who are treated in facilities, mainly HCs, that don’t qualify for EmOC. As in our study, assisted vaginal delivery is the least likely signal function to be reported at HCs [28]. This emphasises the need to focus attention on HCs in improving EmOC coverage.

Our findings also show that the relative importance of coverage, equity and quality gaps may be quite variable across countries, and therefore a multi-dimension analytical approach is necessary in order to customize policies and interventions. The reported plans of action in the five project districts reflect how priority actions may be different in situations that may have looked similar at a more traditional analysis based, for example, only on mortality and coverage assessments.

The extent to which low quality hampers the achievement of the desired health outcomes in spite of good access to institutional delivery is striking and confirms the need to ensure effective coverage of perinatal care and not just access to care. Similar conclusions were recently drawn by large scale projects aimed at improving institutional deliveries in other high-burden countries [31]. On the contrary, while better quality of care reflects in much better mortality indicators for both mothers and babies in Wolisso hospital than in the other two hospitals, good quality care provided to less than 20% of women is clearly not enough to reduce overall maternal and neonatal mortality and morbidity at population level, which remains high at both district and country level [32].

As shown in previous quality assessments [27], there may be gaps in infrastructure, commodities and staff that need to be addressed at system level, but several quality gaps appear to be manageable with the existing resources at local level. On the same line, it is worth noting that scoring was generally higher in areas such as availability of laboratory services and drugs, indicating that shortage of commodities is not the only reason for low quality of care.

Our study suffered from a few limitations. Our assessment of equity relied on comparing data collected from service users with historical data collected through household surveys. This method assumes that the wealth status in the population is constant between the time of the household surveys and the time of our surveys. Although some changes might have occurred, we believe the use of household assets, which reflect wealth that has accumulated over a long period of time, minimised this effect. Measuring coverage relied on routine health data which may not be of good quality [33]; this might have influenced our results. We tried to minimize this effect by triangulating data from different sources including patient files, registers and monthly report summaries. The number of stillbirths and neonatal deaths with missing data on birth weight or timing of deaths might have resulted into underestimation of intrapartum and very early neonatal death rate, especially at Tosamaganga hospital. Assuming that all the cases with missing data were eligible for inclusion, the revised values for intrapartum and very early neonatal death rate would be 1.1%, 2.3% and 2.2% instead of 1.0%, 2.3% and 1.5% for Wolisso, Aber and Tosamaganga hospitals, respectively. In Iringa District, for geographical reasons, some women might be delivering at Iringa Regional Hospital which is located 18 km away from Tosamaganga Hospital. It is therefore possible that we underestimated caesarean section coverage, the proportion of all births in EmOC facility and the met need for EmOC services. This observation may also partly explain the shape of the equity graph for Iringa: some women in the highest wealth group may have delivered at the regional hospital. However, results would not have changed considerably and, most important, the main conclusions would have remained unchanged.

Our findings provide evidence that a three-dimensional approach to improving maternal and neonatal health is necessary, otherwise the desired health outcomes may not be achieved either because most of the population has no access to health care or because once access is ensured, the quality of care provided is poor. Although this evidence is not surprising, so far too little has been done to comprehensively assess coverage, equity and quality of services for mothers and newborn babies so as to identify, address and mitigate the existing gaps in all these dimensions.

The project has now moved to implementing actions to address the causes of the gaps in coverage, equity and quality which have been identified. Some of the underlying causes pertain to distal determinants and demand issues such as extreme poverty, food insecurity, low education, cultural obstacles to adequate reproductive health and insufficient transport, and therefore need to be addressed by “whole government” policies [34]. Others regard key health system components, from financing to procurement of commodities, to training and deployment of human resources, health information system and delivery modes [34]. Although most of the issues need to be addressed by national governments, several actions can be effectively taken at the local level, in collaboration with local communities, health authorities, managers and health professionals [35].

## Supporting Information

S1 Table Formulae used to calculate EmOC indicators. ^a^Crude birth rate x district population ^b^ Direct obstetric complications included: Antepartum haemorrhage, postpartum haemorrhage/retained placenta, obstructed/prolonged labour, raptured uterus, postpartum sepsis, severe pre-eclampsia/eclampsia, severe complications of abortion and ectopic pregnancy. ^c^ Intrapartum deaths and neonates with missing birth weight or timing of foetal deaths were excluded from analysis. The number of stillbirth and neonates with unspecified weight or timing of deaths in EmoC facilities were: 13 in Tanzania, 5 in Uganda and 3 in Ethiopia.
(DOCX)

S2 Table Main features of the equity surveys. (DOCX)

S3 Table Assigning scores and weights to selected variables. (DOCX)

S4 Table Reasons for not performing signal functions at health centres. ^a^ Multiple responses allowed. Reasons for not performing signal functions were classified as follows. a. Availability of human resources. 1. Required health workers are not posted to this facility in adequate numbers (or at all). b. Training issues 1. Authorized cadre is available, but not trained 2. Providers lack confidence in their own skills c. Supplies/Equipment Issue 1. Supplies/equipment are not available, not functional, or broken 2. Needed drugs are unavailable d. Management Issues 1. Providers desire compensation to perform this function 2. Providers are encouraged to perform alternative procedures 3. Providers uncomfortable or unwilling to perform procedure for reasons unrelated to training 4. Lack of supervision e. Policy issues- national or facility policies do not allow function to be performed f. No Indication—no client needing this procedure came to the facility during this time period.
(DOCX)
