# Correction: Assessing Coverage, Equity and Quality Gaps in Maternal and Neonatal Care in Sub-Saharan Africa: An Integrated Approach

**DOI:** 10.1371/journal.pone.0130859

**Published:** 2015-06-19

**Authors:** Calistus Wilunda, Giovanni Putoto, Donata Dalla Riva, Fabio Manenti, Andrea Atzori, Federico Calia, Tigist Assefa, Bruno Turri, Onapa Emmanuel, Manuela Straneo, Firma Kisika, Giorgio Tamburlini

The last author’s name is spelled incorrectly. The correct name is: Giorgio Tamburlini.

## References

[1] Wilunda C, Putoto G, Riva DD, Manenti F, Atzori A, Calia F, et al (2015) Assessing Coverage, Equity and Quality Gaps in Maternal and Neonatal Care in Sub-Saharan Africa: An Integrated Approach. PLoS ONE 10(5): e0127827 doi:10.1371/journal.pone.0127827 26000964 10.1371/journal.pone.0127827 PMC4441493

