# Supplementary material for: Assessing Coverage, Equity and Quality Gaps in Maternal and Neonatal Care in Sub-Saharan Africa: An Integrated Approach
Source: PLoS One. 2015 May 22;10(5):e0127827. doi: 10.1371/journal.pone.0127827 (PMC4441493; doi:10.1371/journal.pone.0127827)
Supplement: S1 Table — aCrude birth rate x district population b Direct obstetric complications included: Antepartum haemorrhage, postpartum haemorrhage/retained placenta, obstructed/prolonged labour, raptured uterus, postpartum sepsis, severe pre-eclampsia/eclampsia, severe complications of abortion and ectopic pregnancy. c Intrapartum deaths and neonates with missing birth weight or timing of foetal deaths were excluded from analysis. The number of stillbirth and neonates with unspecified weight or timing of deaths in EmoC facilities were: 13 in Tanzania, 5 in Uganda and 3 in Ethiopia. (DOCX) [file pone.0127827.s001.docx]

S1 Table. Formulae used to calculate EmOC indicators

| **EmOC indicator** | **Formula for calculation** |
| --- | --- |
| 1a. Number of comprehensive EmOC facilities per 500,000 population | (Number of comprehensive EmOC facilities/district population) x 500,000 |
| 1b. Number of basic EmOC facilities per 500,000 population | (Number of basic EmOC facilities/district population) x 500,000 |
| 2. Proportion of all births in EmOC facility | (Number of women giving births in EmOC facility/expected births in the district ^a^) x 100 |
| 3. Met need for EmOC services | [Number of women with direct obstetric complications ^b^ treated in EmOC facility/(expected births in the district x 0.15) ] x 100 |
| 4. Caesarean sections as a proportion of all births | (Number of caesarean sections/expected births in the district) x 100 |
| 5. Direct obstetric case fatality rate in EmOC facility | (Number of maternal deaths from direct obstetric causes in EmOC facilities/Number of women with obstetric complications in EmOC facilities) x 100 |
| 6. Intrapartum and very early neonatal death rate in EmOC facility ^c^ | (Number of intrapartum deaths (≥ 2.5 kg) + very early neonatal deaths (≤ 24 h; ≥ 2.5 kg) in EmOC facilities/Number of women who gave birth in EmOC facilities) x 100 |
| 7. Proportion of maternal deaths due to indirect causes in EmOC facility | (Number of maternal deaths from indirect causes in EmOC facilities/ Number of maternal deaths from all causes in EmOC facilities) x 100 |
