# Supplementary material for: Assessing Coverage, Equity and Quality Gaps in Maternal and Neonatal Care in Sub-Saharan Africa: An Integrated Approach
Source: PLoS One. 2015 May 22;10(5):e0127827. doi: 10.1371/journal.pone.0127827 (PMC4441493; doi:10.1371/journal.pone.0127827)
Supplement: S2 Table — (DOCX) [file pone.0127827.s002.docx]

**S2 Table. Main features of the equity surveys**

| **Feature** | **Ethiopia** | **Uganda** | **Tanzania** |
| --- | --- | --- | --- |
| Source of data | 2011 Demographic and Health Survey (Oromiya Region) | 2006 Demographic and Health Survey (Northern Region) | 2010 Tanzania Demographic and Health Survey (Iringa Region) |
| Proxy wealth variables | Source of drinking water, roof material, ownership of a bed; a mobile phone ; a table; and a radio | Roof material, wall material, woman’s education and ownership of table; a bed; and a radio; | Roof material, floor material and ownership of a mobile phone; a television set; a bank account and electricity supply. |
| Validity and reliability | Rho: 0.908;  Kappa: 0.52, 95% CI 0.49 -0.55 | Rho: 0.904;  Kappa: 0.549, 95% CI 0.512 to 0.586 | Rho: 0.95;  Kappa: 0.611, 95% CI: 0.550 to 0.672 |
| Facilities involved | 1 hospital  7 health centres | 1 hospital  6 Health centres | 1 hospital |
| Data collection period | May to September 2012 at the hospital and September 2012 to February 2013 at HCs | December 2011 to March 2012 | January to June 2012 |
