# Supplementary material for: Assessing Coverage, Equity and Quality Gaps in Maternal and Neonatal Care in Sub-Saharan Africa: An Integrated Approach
Source: PLoS One. 2015 May 22;10(5):e0127827. doi: 10.1371/journal.pone.0127827 (PMC4441493; doi:10.1371/journal.pone.0127827)
Supplement: S3 Table — (DOCX) [file pone.0127827.s003.docx]

**S3 Table. Assigning scores and weights to selected variables**

| 1. **Oyam District, Uganda** | | | | | |
| --- | --- | --- | --- | --- | --- |
| **Variable** | **Categories** | **Raw score** | **Rescaled score** | **Weight** | **Weighted score** |
| Roof material | Thatched/mud | 1 | 0 | 6 | 0 |
|  | Iron sheets/asbestos | 2 | 1 |  | 6 |
| Wall material | Thatched/straw | 1 | 0 | 5 | 0 |
|  | Mud and poles | 2 | 0.25 |  | 1.25 |
|  | Un-burnt bricks | 3 | 0.5 |  | 2.5 |
|  | Burnt bricks with mud | 4 | 0.75 |  | 3.75 |
|  | Cement blocks/burnt bricks with cement | 5 | 1 |  | 5 |
| Has a table | No | 1 | 0 | 4 | 0 |
|  | Yes | 2 | 1 |  | 4 |
| Has a bed | No | 1 | 0 | 3 | 0 |
|  | Yes | 2 | 1 |  | 3 |
| Radio | No | 1 | 0 | 2 | 0 |
|  | Yes | 2 | 1 |  | 2 |
| Education attainment | No education | 1 | 0 | 1 | 0 |
|  | Incomplete primary | 2 | 0.25 |  | 0.25 |
|  | Complete primary | 3 | 0.5 |  | 0.5 |
|  | Incomplete secondary | 4 | 0.75 |  | 0.75 |
|  | Higher | 5 | 1 |  | 1 |
| 1. **Wolisso, Goro and Wonchi districts, Ethiopia** | | | | | |
| Source of drinking water | Unprotected well/spring/river/ dam/lake/ponds/ stream /canal/irrigation channel | 1 | 0 | 6 | 0 |
|  | Borehole/protected well | 2 | 0.33 |  | 1.98 |
|  | Public tap/standpipe | 3 | 0.67 |  | 4.02 |
|  | Piped into dwelling/plot/yard | 4 | 1 |  | 6 |
| Main roof material | No roof/Thatch/leaf/mud/plastic sheets/Reed/ Bamboo/ Wood planks/ Cardboard | 1 | 0 | 5 | 0 |
|  | Corrugated iron/wood/asbestos/timber/concrete | 2 | 1 |  | 5 |
| Bed with cotton/ sponge/ spring mattress | No | 1 | 0 | 4 | 0 |
|  | Yes | 2 | 1 |  | 4 |
| Has mobile telephone | No | 1 | 0 | 3 | 0 |
|  | Yes | 2 | 1 |  | 3 |
| Table | No | 1 | 0 | 2 | 0 |
|  | Yes | 2 | 1 |  | 2 |
| Has radio | No | 1 | 0 | 1 | 0 |
|  | Yes | 2 | 1 |  | 1 |

**S3 Table (continued)**

1. **Iringa district, Tanzania**

| **Variable** | **Categories** | **Raw score** | **Rescaled score** | **Weight** | **Weighted score** |
| --- | --- | --- | --- | --- | --- |
| Main floor material | Earth, sand, dung | 1 | 0 | 6 | 0 |
|  | Ceramic tiles/cement/carpet | 2 | 1 |  | 6 |
| Has television | No | 1 | 0 | 5 | 0 |
|  | Yes | 2 | 1 |  | 5 |
| Has electricity | No | 1 | 0 | 4 | 0 |
|  | Yes | 2 | 1 |  | 4 |
| Has a mobile telephone | No | 1 | 0 | 3 | 0 |
|  | Yes | 2 | 1 |  | 3 |
| Owns a bank account | No | 1 | 0 | 2 | 0 |
|  | Yes | 2 | 1 |  | 2 |
| Main roof material | Grass/thatch/mud | 1 | 0 | 1 | 0 |
|  | Iron sheets | 2 | 1 |  | 1 |
