# Supplementary material for: Assessing Coverage, Equity and Quality Gaps in Maternal and Neonatal Care in Sub-Saharan Africa: An Integrated Approach
Source: PLoS One. 2015 May 22;10(5):e0127827. doi: 10.1371/journal.pone.0127827 (PMC4441493; doi:10.1371/journal.pone.0127827)
Supplement: S4 Table — a Multiple responses allowed. Reasons for not performing signal functions were classified as follows. a. Availability of human resources. 1. Required health workers are not posted to this facility in adequate numbers (or at all). b. Training issues 1. Authorized cadre is available, but not trained 2. Providers lack confidence in their own skills c. Supplies/Equipment Issue 1. Supplies/equipment are not available, not functional, or broken 2. Needed drugs are unavailable d. Management Issues 1. Providers desire compensation to perform this function 2. Providers are encouraged to perform alternative procedures 3. Providers uncomfortable or unwilling to perform procedure for reasons unrelated to training 4. Lack of supervision e. Policy issues- national or facility policies do not allow function to be performed f. No Indication—no client needing this procedure came to the facility during this time period. (DOCX) [file pone.0127827.s004.docx]

**S4 Table: Reasons for not performing signal functions at health centres**

| **Signal function** | **No. of HCs that did not perform signal function** | **Number of HCs that did not perform signal function due to ^a^:** | | | | | |
| --- | --- | --- | --- | --- | --- | --- | --- |
|  |  | **Lack of human resources** | **Training issues** | **Supplies/ equipment** | **Management issues** | **Policy issues** | **No indication** |
| Parenteral Oxytocics | 1 | 0 | 0 | 0 | 0 | 0 | 1 |
| Manual removal of placenta | 7 | 1 | 2 | 0 | 0 | 0 | 4 |
| Neonatal resuscitation | 6 | 0 | 2 | 3 | 0 | 0 | 1 |
| Parenteral antibiotics | 5 | 0 | 0 | 0 | 0 | 0 | 5 |
| Removal of retained products | 7 | 0 | 3 | 1 | 0 | 0 | 6 |
| Assisted vaginal delivery | 19 | 1 | 11 | 10 | 2 | 1 | 7 |
| Parenteral anticonvulsants | 14 | 1 | 3 | 4 | 2 | 0 | 11 |
| HCs mentioning any of the reasons |  | 3 | 21 | 18 | 4 | 1 | 35 |
